# Novel evidence for complement system activation in chick myopia and hyperopia models: a meta-analysis of transcriptome datasets

**DOI:** 10.1038/s41598-017-10277-2

**Published:** 2017-08-29

**Authors:** Nina Riddell, Sheila G. Crewther

**Affiliations:** Department of Psychology and Counselling, School of Psychology and Public Health, La Trobe University, Melbourne, Victoria 3086 Australia

## Abstract

Myopia (short-sightedness) and hyperopia (long-sightedness) occur when the eye grows too long or short, respectively, for its refractive power. There are currently approximately 1.45 billion myopes worldwide and prevalence is rising dramatically. Although high myopia significantly increases the risk of developing a range of sight-threatening disorders, the molecular mechanisms underlying ocular growth regulation and its relationship to these secondary complications remain poorly understood. Thus, this study meta-analyzed transcriptome datasets collected in the commonly used chick model of optically-induced refractive error. Fifteen datasets (collected across five previous studies) were obtained from GEO, preprocessed in Bioconductor, and divided into 4 conditions representing early (≤1 day) and late (>1 day) myopia and hyperopia induction. Differentially expressed genes in each condition were then identified using Rank Product meta-analysis. The results provide novel evidence for transcriptional activation of the complement system during both myopia and hyperopia induction, and confirm existing literature implicating cell signaling, mitochondrial, and structural processes in refractive error. Further comparisons demonstrated that the meta-analysis results also significantly improve concordance with broader omics data types (i.e., human genetic association and animal proteomics studies) relative to previous transcriptome studies, and show extensive similarities with the genes linked to age-related macular degeneration, choroidal neovascularization, and cataract.

## Introduction

Recent world-wide increases in the prevalence of myopia (short-sightedness) suggest that environmental and/or lifestyle factors are extraordinarily important to the underlying pathogenesis of this complex disorder^1^. Myopia typically results when excessive axial growth causes a mismatch between the eyes refractive power and its length^2^. By comparison, hyperopic (long-sighted) eyes are usually small^3^. Environmentally-driven changes to ocular axial growth and consequent refractive state have been investigated using animal models, in which rearing with negatively powered defocusing lenses or form deprivation occluders increases eye growth resulting in myopia, while rearing with positive lenses induces growth arrest and hyperopia (long-sightedness)^4^. Although early studies in these models were primarily limited to hypothesis-driven approaches (for example refs 5–8) the recent availability of microarray and RNA-sequencing technologies has enabled a number of discovery-driven investigations of gene expression in the posterior eye. To date, eleven such transcriptome studies have been published encompassing a range of species (primate, mouse, and chick), platforms (commercial microarrays, custom microarrays, and RNA-sequencing), optical manipulations (lens-induced myopia; LIM, form deprivation occlusion myopia; FDM, lens-induced hyperopia; LIH, and FDM recovery) and tissue samples (retina, RPE, choroid, sclera)^9–19^. These studies have offered many insights into the biology of optically-induced ocular growth changes, however, the key expression responses underlying refractive error induction remain unclear as >1500 genes have been implicated with poor cross-study replication^20, 21^.

Thus, the present study aimed to use meta-analysis techniques to generate a more reliable understanding of the generalized transcriptome responses underlying refractive error induction in animal models. Studies were considered for inclusion in the meta-analysis if they used commercially available microarray chips or RNA-sequencing to profile mRNA expression in the posterior eye of animal models of optically-induced myopia or hyperopia and matched no lens controls. Based on these criteria, five of the eleven available transcriptome studies were excluded because they lacked a no lens control comparison^10, 12^, used custom microarray chips with limited genome coverage^9, 18^, or profiled miRNA rather than mRNA^19^. Five of the remaining six studies profiled gene expression in chick retinal, RPE, and/or choroidal tissue^13–17^. The final study analyzed scleral transcriptome responses in the form deprivation mouse model^11^. This latter study was excluded because it differed from the others both in the tissue profiled (sclera rather than retina/RPE/choroid) and model species (all other included studies were conducted in chick, the most widely used animal model for refractive error research).

The remaining five chick studies profiled a range of posterior ocular tissues (comprised of varying combinations of retina, RPE, and/or choroid), induction time-points (ranging from 6 hours to 3 days), and optical manipulations (myopia induction via occlusion or negative lenses, or hyperopia induction via positive lenses). Despite this, published comparisons (e.g. see supplementary materials Stone *et al*.^15^ and Riddell *et al*.^13^) have identified a number of common findings across the studies, including similarities between studies profiling very different tissue compositions such as retina/RPE and amacrine cells. This suggests that, although the expression data must to some extent reflect the specific cell types profiled, at least a subset of responses during refractive error induction are sufficiently generalized or sufficiently strong to be measurable regardless of the exact tissue composition. This pattern of results is in accordance with most theories of environmentally-driven ocular growth which postulate a signal or cascade of signals that propagate across the posterior eye (presumably inducing related expression responses from multiple cell types)^4, 22–24^. Likewise, there is evidence for extensive similarities in the molecular response to FDM and LIM^21^.

Based on these past observations, datasets profiling different tissue compositions (retina, RPE, and choroid) and myopia-inducing manipulations (LIM and FDM) were not separated in the meta-analysis. We did, however, separate datasets profiling early (≤1 day) and later (>1 day) induction time-points (as described previously^21^). This decision was based on past research suggesting that early and late expression responses are dissimilar in chick^14, 15^, in line with the theoretical expectation that the biological processes involved in the onset and progression of refractive change are likely to be active at early time-points, while secondary responses to prolonged lens-wear may predominate at later time-points^15, 20^.

The separation of datasets into early and late categories resulted in four meta-analysis conditions: early myopia (4 datasets), late myopia (4 datasets), early hyperopia (4 datasets), and late hyperopia (3 datasets). The datasets included in each of these conditions were heterogeneous, encompassing a range of circadian phases, developmental ages, tissue types, control conditions (fellow eye or separate no lens), and exact optical manipulations (occlusion or lenses of various powers). We considered this heterogeneity to be a strength in the analysis design, as genes showing differential expression across these diverse datasets are presumably less likely to reflect these confounding influences. Given the diversity of the datasets, we chose to use the non-parametric rank product meta-analysis method that transforms expression values into ranks to identify consistently highly ranked genes across replicate experiments (i.e., genes that are consistently up- or down-regulated)^25^. The Rank Product approach has higher sensitivity and selectivity than *t-*based meta-analysis methods, particularly when sample sizes are small and data is heterogeneous^26^.

## Results

### Initial evaluation of the meta-analysis results

**Figure 1:**
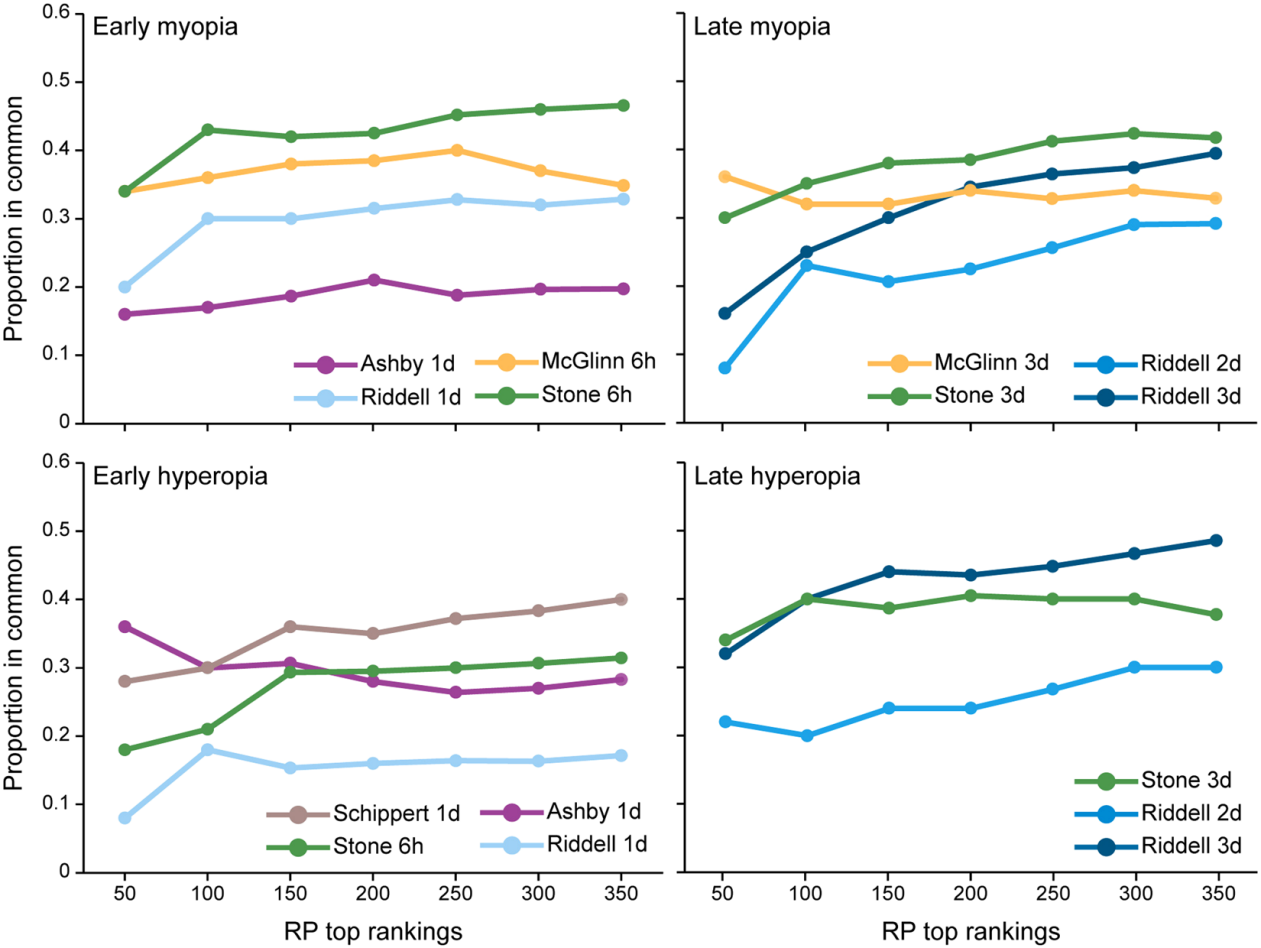
Correspondence at the top (CAT) plots showing the contribution of each dataset to the meta-analysis findings. These plots evaluate the consensus between top ranking genes from each individual dataset and top ranking genes from each meta-analysis condition (early and late myopia and hyperopia). The x-axis is top ranking genes based on the rank product analyses and the y-axis is the proportion of genes in common with the meta-analysis results.

Datasets profiling gene expression in the chick retina, RPE, and/or choroid during myopia (8 datasets) and hyperopia (7 datasets) induction were split into early and late time-points, and differentially-expressed genes in each condition were then identified using rank product meta-analysis. Fifty-nine and 189 genes were differentially-expressed in early and late myopia induction conditions, respectively (Supplementary Table S1). Twenty-seven and 136 genes were differentially-expressed in early and late hyperopia induction conditions (Supplementary Table S2). Correspondence at the top (CAT) plots were used to assess the contribution of each dataset to the meta-analysis findings. These plots show the proportion of top ranked genes from each individual dataset present in the meta-analysis results as a function of list size. As shown in Fig. 1, no individual dataset disproportionately accounted for the meta-analysis findings.

**Figure 2:**
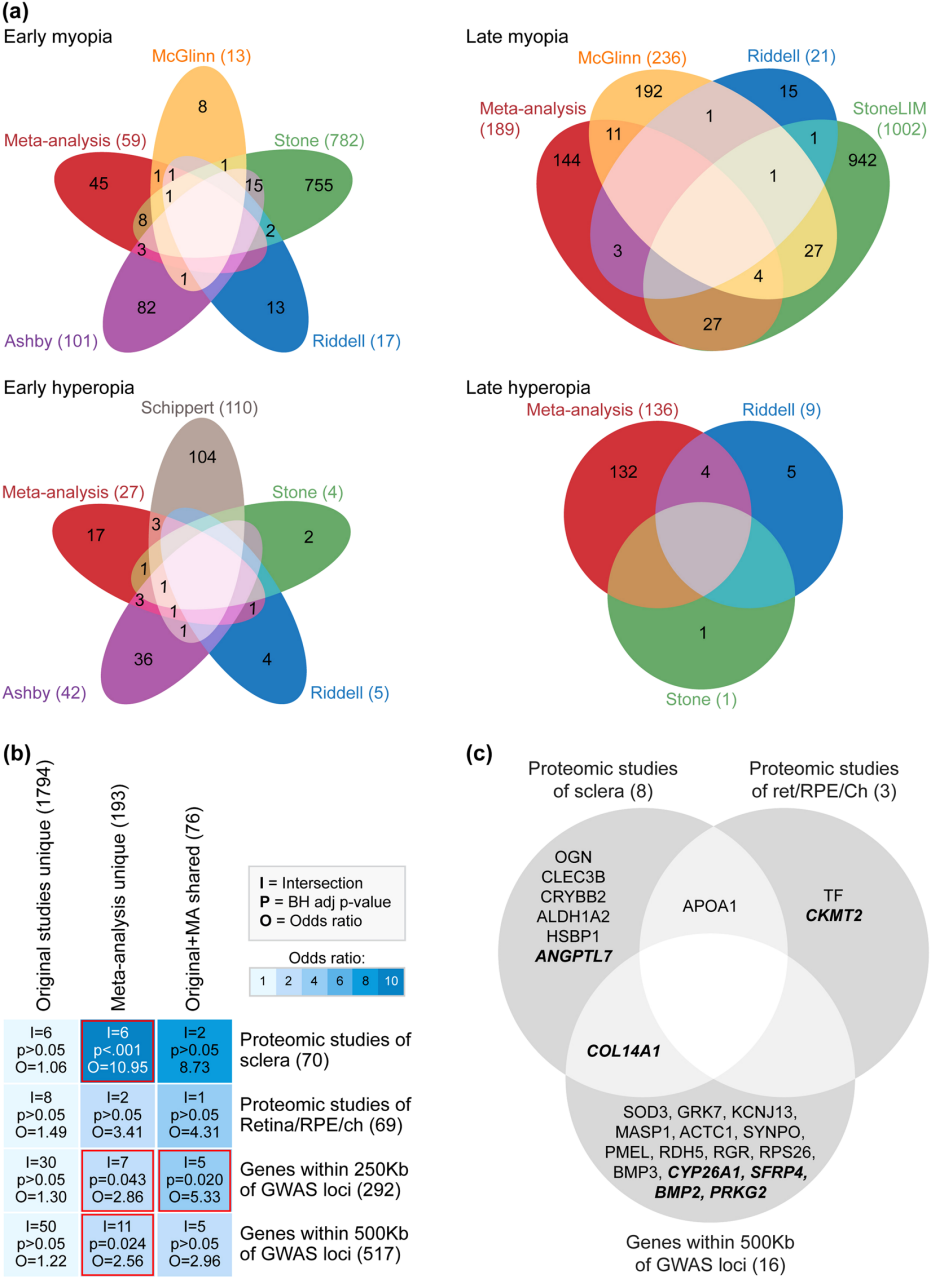
The meta-analysis results show improved concordance with previous animal proteomic and human GWAS findings. (**a**) Venn diagrams comparing differentially-expressed genes from each meta-analysis (pfp < 0.05) with differentially-expressed genes from the individual studies that were meta-analyzed (as per the original authors’ statistical criteria). Note that most of the genes identified in the rank product meta-analyses were unique (i.e., not classified as differentially-expressed in the original transcriptome studies). (**b**) Heat map comparing the overlap of original transcriptome study results and rank product meta-analysis results with the genes implicated in previous GWAS and proteomics studies of refractive error. The shared category includes genes implicated in both the rank product meta-analyses and the individual studies included in the meta-analyses. Meta-analysis and original study categories include the remaining genes that were uniquely implicated in either the rank product meta-analysis or the original studies. The number of genes implicated in each list is shown in parenthesis after the list name. List intersections (i.e., the number of overlapping genes), *Benjamini–Hochberg* adjusted *p*-values, and odds ratios are superimposed on the heat map grid. Statistically significant overlaps are highlighted in red. Note that, although there were generally more commonalities in total between the original transcriptome study findings and the proteomics/GWAS results, the list of genes implicated in the original studies was very large resulting in low statistical significance and odds ratios. Supplementary Table S3 provides further details on all of the overlapping genes. (**c**) Venn diagram showing the rank product meta-analysis genes that were also implicated in previous GWAS or proteomics studies (i.e., all overlapping genes from the right two columns of Fig. 2b). Shared findings are shown in bold italic font.

Most of the meta-analysis findings were unique (i.e., the genes differentially-expressed in the meta-analysis were not differentially-expressed in any of the individual transcriptome studies that were meta-analyzed; see Fig. 2a). To test the utility of these new results, we compared both the original transcriptome findings and the meta-analysis findings with the genes and proteins implicated in human Genome-Wide Association Studies (GWAS) and animal proteomics studies of refractive error. As reported previously^21^, the individual studies included in the meta-analysis implicated a number of genes that were also differentially-expressed in animal proteomics studies of refractive error and/or located within 500 kilobases (Kb) of human GWAS ocular axial length or refractive error loci (Fig. 2b). The meta-analysis replicated some of these results, and identified 18 new commonalities with GWAS and proteomics studies (Fig. 2b,c). Because the list of genes implicated in the meta-analysis was much smaller than the list of genes implicated in the individual transcriptome studies, the meta-analysis results showed more statistically significant concordance with these broader omics data types.

### Pathway over-representation

**Figure 3:**
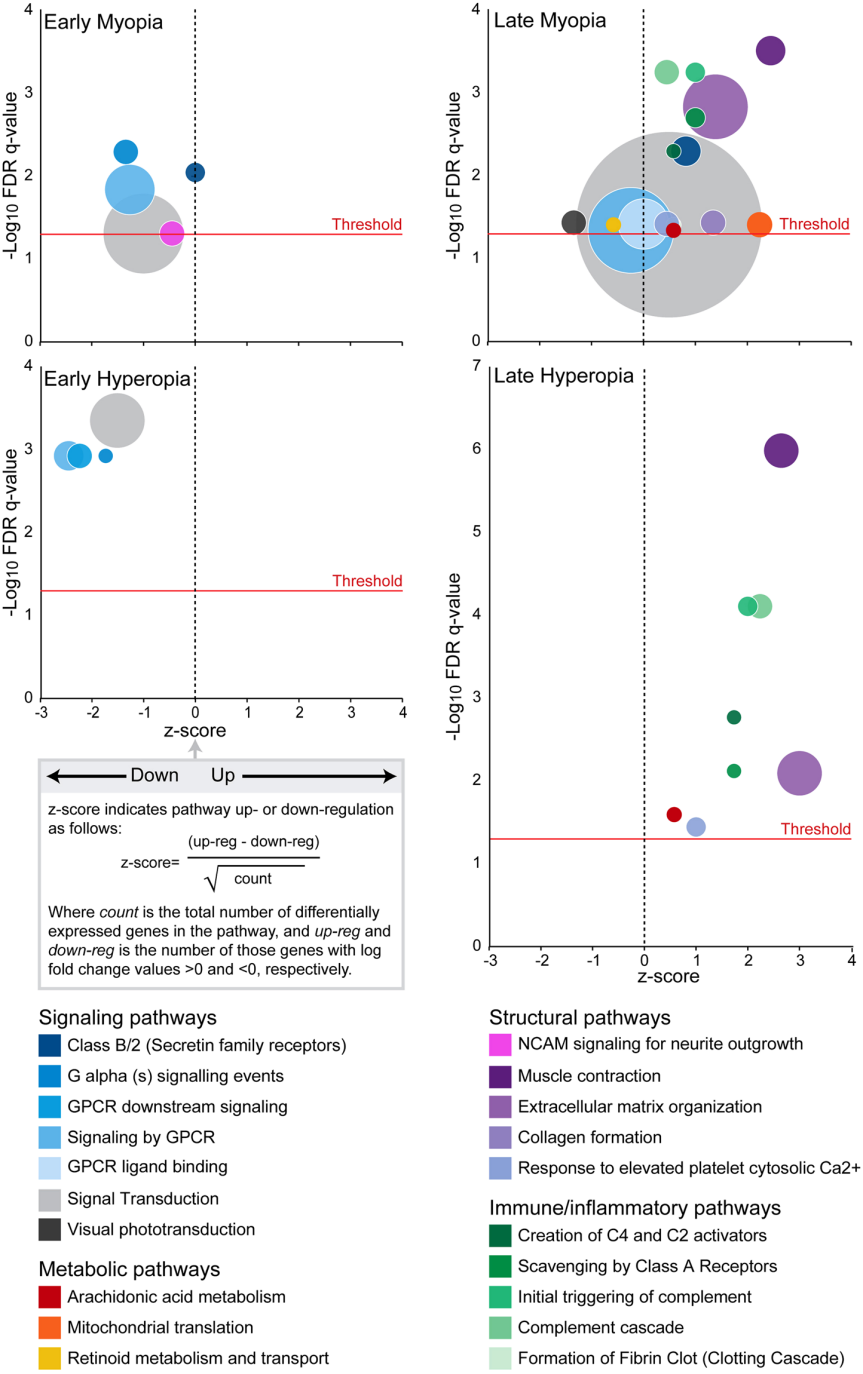
Bubble plots showing over-represented Reactome pathways in each meta-analysis condition. The size of each bubble represents the number of genes contributing to pathway over-representation (ranging from 3–37), and the z-score indicates whether genes from the pathway were primarily up-regulated (positive z-score) or down-regulated (negative z-score). The FDR q-value cut-off of 0.05 is indicated by the ‘threshold’ line. Detailed over-representation results are provided in Supplementary Table S4.

After initial evaluations, we tested the differentially-expressed genes in each meta-analysis condition for over-representation of Reactome pathways. Several signaling pathways (particularly those related to G-protein coupled signaling) were over-represented in the genes differentially-expressed in the early myopia and hyperopia datasets (Fig. 3). In addition, genes from the NCAM signaling for neurite outgrowth pathway were over-represented in the early myopia data only. A wider range of processes were implicated in the late myopia and hyperopia, with genes from immune/inflammation (complement-coagulation cascade and class A scavenging receptors), tissue structure (extracellular matrix and muscle contraction), and arachidonic acid metabolism over-represented in both datasets (Fig. 3). Several signal transduction pathways were also implicated in the late myopia dataset, along with genes involved in phototransduction, retinoid metabolism, and mitochondrial translation. Detailed over-representation results are provided in Supplementary Table S4.

### Common gene and pathway findings across conditions

**Figure 4:**
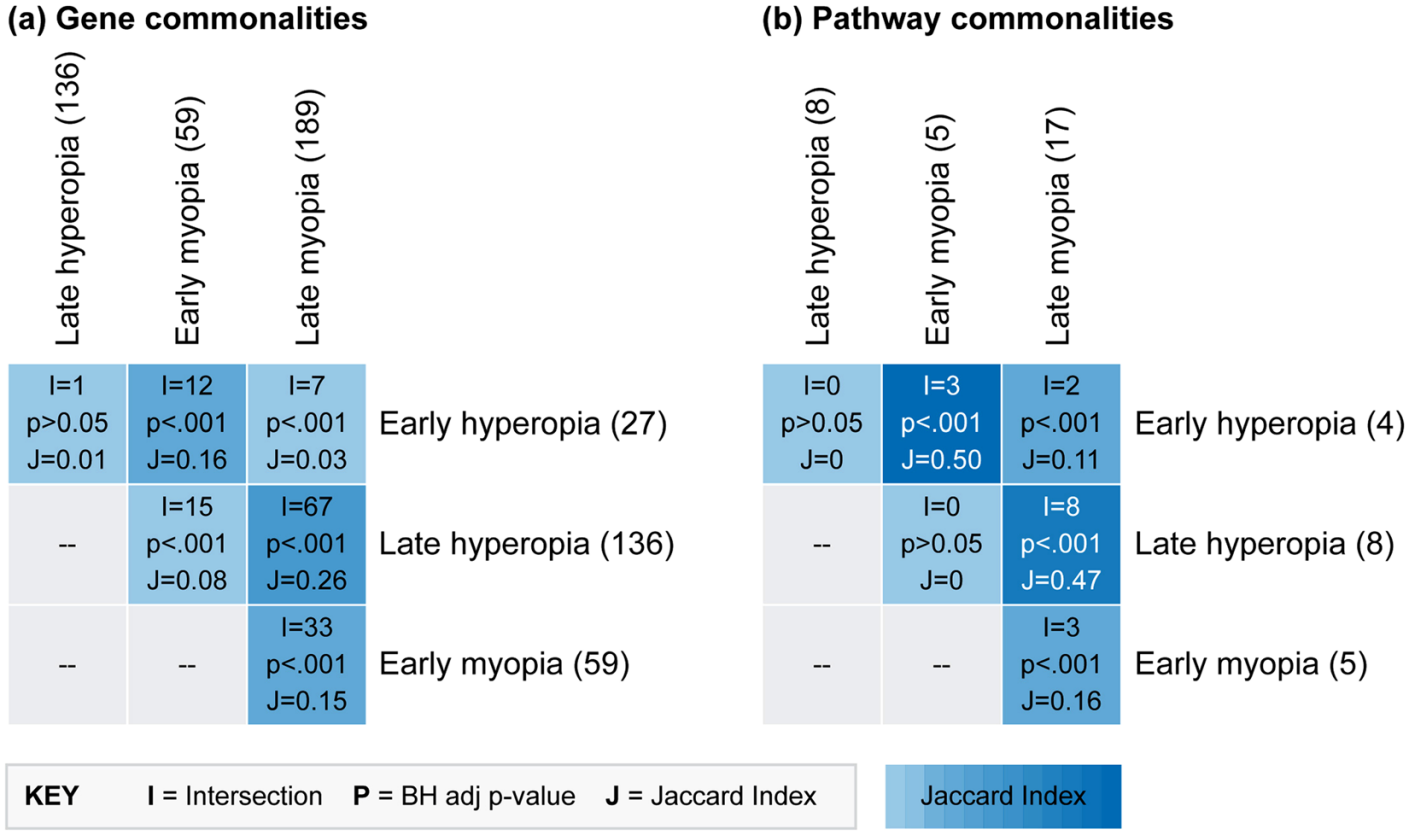
Common gene and pathway findings across meta-analysis conditions. Heat maps show overlap in (**a**) the genes differentially-expressed in each meta-analysis condition and (**b**) the pathways over-represented in each meta-analysis condition. The number of genes/pathways implicated in each meta-analysis condition is shown in parenthesis after the condition name. List intersections (i.e., the number of overlapping genes/pathways), *Benjamini–Hochberg* adjusted *p*-values, and Jaccard similarity indices are superimposed on the heat map grid. Note that the genes and pathways implicated in myopia and hyperopia conditions at each time-point are more similar than the genes implicated within each growth condition over time. Supplementary Table S5 provides a detailed list of the overlapping gene and pathway findings.

We next assessed the degree of overlap between early and late myopia and hyperopia conditions at both the gene and pathway level. A large number of genes were differentially-expressed in multiple meta-analyses, with all possible conditions except early and late hyperopia showing more overlap then expected (*p* < 0.001) given the number of genes profiled (Fig. 4a). Most notably, the genes implicated in myopia and hyperopia conditions at each time-point were more similar than the genes implicated within each growth condition over time (see Jaccard Similarity indices in Fig. 4a). This pattern was also evident at the pathway level, where Jaccard similarity indices were 0.5 and 0.47 for early and late time-points, respectively (Fig. 4b). Full details of the overlap in gene and pathway findings are provided in Supplementary Table S5.

**Figure 5:**
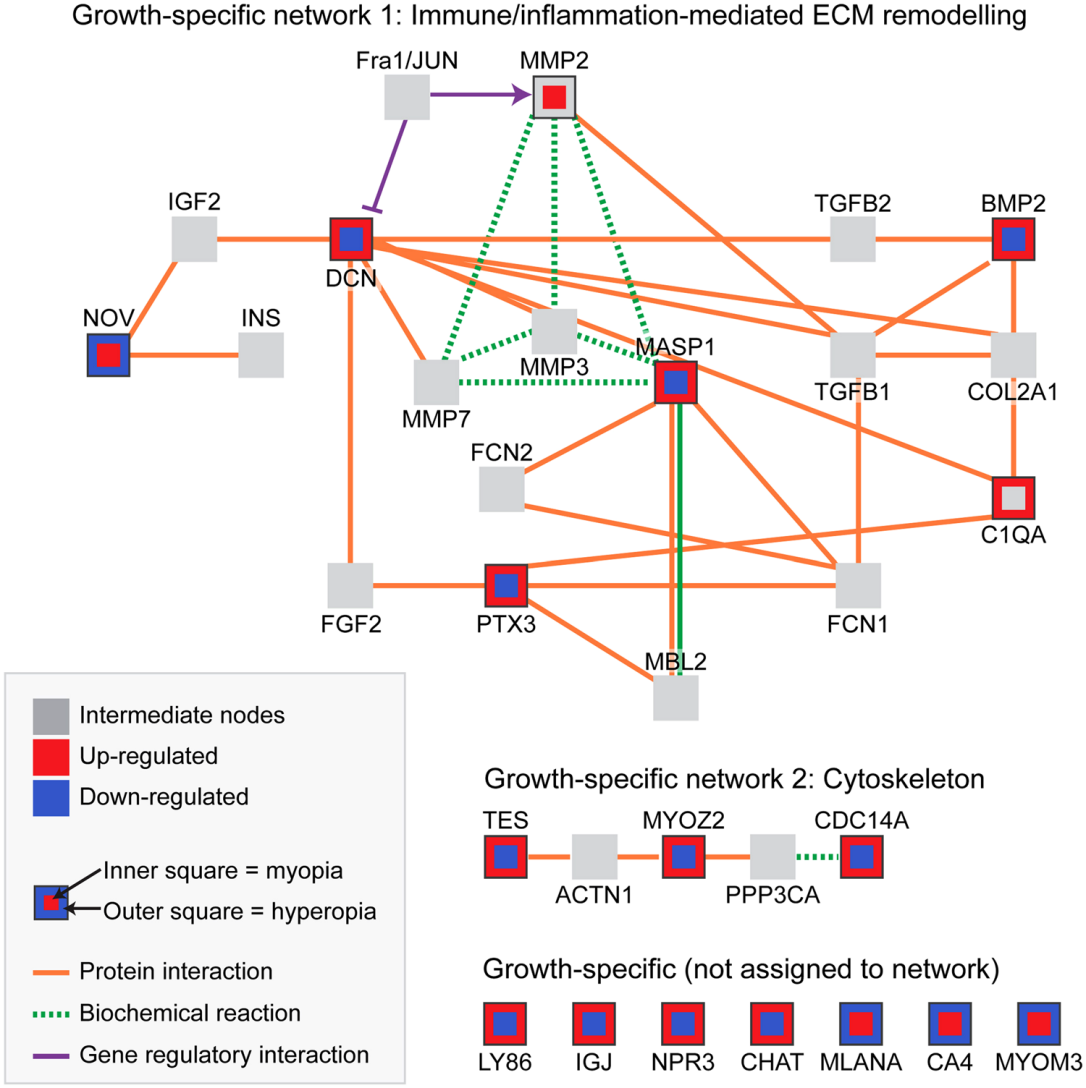
Network analysis demonstrates interactions between genes showing growth-specific expression patterns. The ConsensusPathDB Induced Network Module Analysis aims to connect a list of seed genes (in this case, the 15 genes showing growth-specific expression patterns) via different types of interactions (protein interactions, biochemical interactions, or gene regulatory interactions). Connections are made directly, or via an intermediate node (shown in grey). Eight of the 15 growth-specific genes were assigned to two highly connected protein networks involved in immune/inflammation-mediated extracellular matrix remodeling and cytoskeletal processes. The remaining 7 growth-specific genes are shown at the bottom of the image. Note that intermediate nodes MMP2 and C1QA were also differentially expressed in the meta-analysis, but not in a growth-specific manner. Information for each interaction in the networks is provided in Supplementary Table S7.

The genes underlying these overlapping results were primarily differentially-expressed in a growth non-specific manner, with just 15 genes displaying growth-specific profiles (i.e., concurrent up-regulation during myopia induction and down-regulation during hyperopia induction, or visa versa; Fig. 5). Induced network module analysis was used to identify relationships between these 15 growth-specific genes, and to predict additional ‘intermediate’ genes or proteins that may be involved in growth-specific processes. This analysis revealed that 5 of the 15 growth-specific genes encoded proteins that form a highly connected network involved in immune/inflammation-mediated extracellular matrix (ECM) remodeling. An additional 3 growth-specific genes encoded proteins involved a network related to cytoskeletal processes (Fig. 5). Given their interconnectivity with growth-specific genes, the intermediate genes from these two networks are good candidates for involvement in growth-specific processes (or refractive error phenotypes more broadly). Indeed, 2 of the 16 intermediate nodes (C1QA and MMP2) were also differentially-expressed in the meta-analysis (though not in a growth-specific manner). Moreover, approximately 50% of the intermediate genes have been implicated in previous targeted studies of myopia development (see Supplementary Table S6).

### Comparison with the genes implicated in common ocular pathologies

We next compared the differentially expressed genes from each meta-analysis condition with the genes previously linked to disorders for which myopia or hyperopia are risk factors. Primary open angle glaucoma (POAG), choroidal neovascularization (CNV) and cataract were chosen for comparison because of their link with myopia^27, 28^. Sub-clinical age-related macular degeneration (AMD) and AMD itself were chosen because of their link with hyperopia^29, 30^.

**Figure 6:**
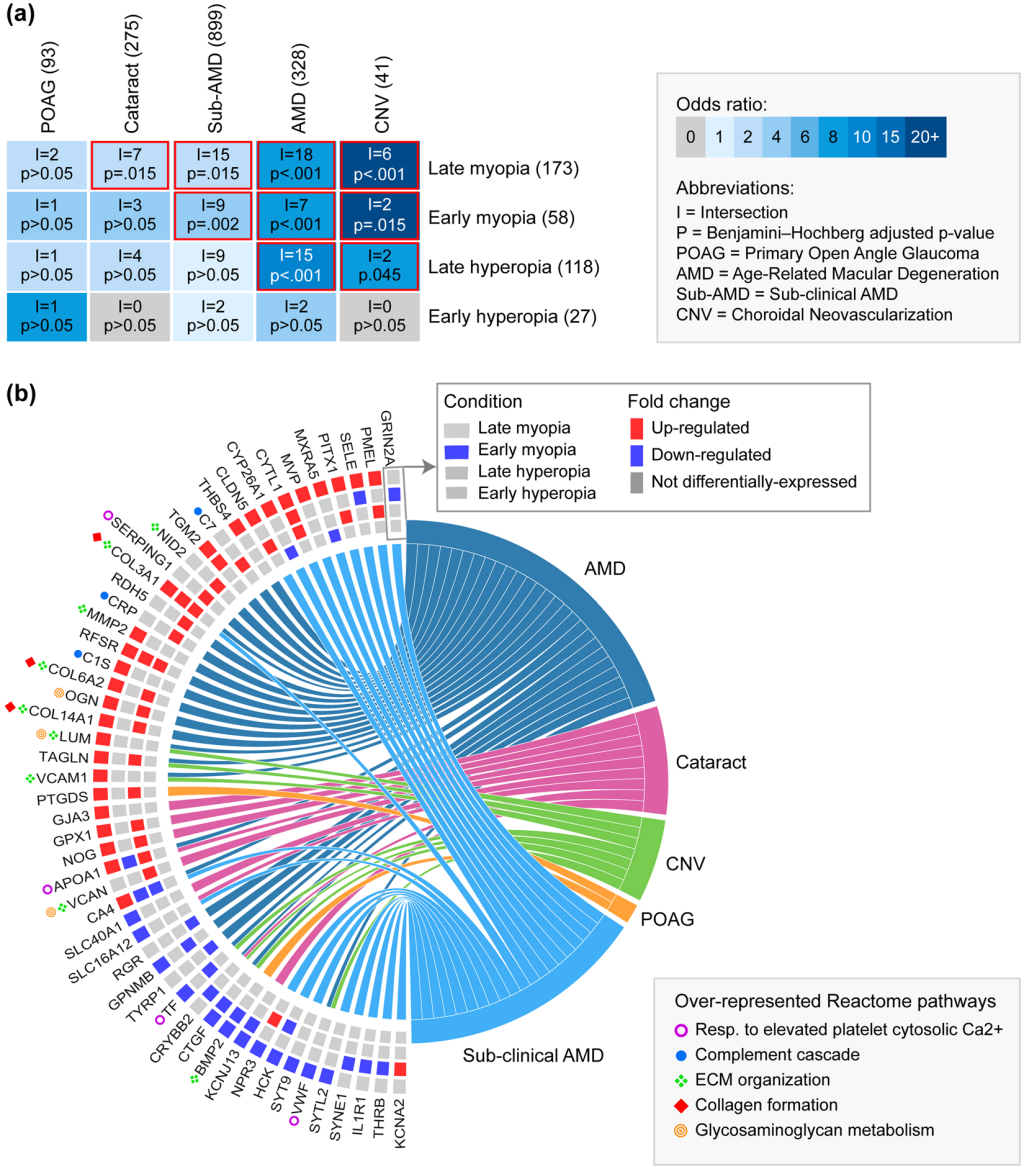
Many differentially-expressed genes from the meta-analysis have previously been associated with ocular pathologies. (**a**) Heat map showing overlap between the genes differentially-expressed in each meta-analysis condition and the genes associated with POAG, cataract, sub-clinical AMD, AMD, and CNV. The number of genes associated with each meta-analysis condition and ocular pathology is shown in parenthesis after the list name. List intersections (i.e., the number of overlapping genes) and *Benjamini–Hochberg* adjusted *p*-values are superimposed on the grid. Statistically significant overlaps are highlighted in red. (**b**) Chord diagram showing genes that were both differentially-expressed in the meta-analysis and associated with an ocular pathology (i.e., all intersecting genes from Fig. 5a). Rectangles on the left indicate whether a gene was differentially expressed in early or late myopia or hyperopia conditions. Left-right connections indicate gene associations with ocular pathologies. Over-represented Reactome pathways for the list of meta-analysis/pathology gene commonalities are shown on the bottom right. Symbols preceding gene names in the chord diagram indicate gene contributions to these pathway over-representations.

As shown in Fig. 6a, the genes differentially expressed in early and late myopia overlapped significantly with the genes associated with sub-clinical AMD, AMD, and CNV. In addition, the genes differentially-expressed in late myopia showed significant similarities with those previously linked to cataract development (Fig. 6a). Although there were fewer similarities evident in the hyperopia conditions, the genes differentially expressed in late hyperopia did overlap significantly with both AMD and CNV associated genes. These gene commonalities as a whole were enriched for pathways related to extracellular structure (ECM organization, collagen formation, glycosaminoglycan metabolism) and the complement-coagulation system (complement cascade, response to elevated platelet cytosolic Ca2^+^; Fig. 6b).

## Discussion

We conducted a rank product meta-analysis of differential gene expression in transcriptome datasets profiling chick retina, RPE, and/or choroid during refractive error induction. The analysis identified a refined list of differentially-expressed genes that shows better concordance relative to past transcriptome studies with both the proteins implicated in animal proteomic studies of refractive error and the candidate genes near human GWAS refractive error loci. We found that gene expression responses during myopia and hyperopia induction were highly similar at both early (≤1 day) and late (>1 day) time-points, and identified just 15 genes showing growth-specific expression profiles.

The early time-point in both growth conditions was characterized by differential-expression of genes involved in a range of signal transduction processes (particularly G-protein coupled receptor signaling), including many genes implicated in previous research (e.g. VIP^31, 32^, GCG^33, 34^, EGR1^5, 35^, and BMP2^36^). At the later time-point, both growth conditions were primarily characterized by differential expression of genes involved in remodeling of cellular and extracellular structure, immune and inflammatory processes, mitochondrial translation, and oxidative defense. Although differential-expression of structural genes is expected given the well-documented morphological features of refractive errors, the involvement of immune, mitochondrial, and oxidative defense genes in both growth conditions at late time-points is notable.

Most of the implicated immune genes were from the complement-coagulation pathway. This pathway has long been known for its role in inducing immune and inflammatory responses, however, recent research has also implicated complement in the promotion of cellular homeostasis (e.g. via metabolic reprogramming)^37^. A diverse range of complement-related genes were differentially-expressed, with components and regulators of both the classical and alternate pathways up-regulated in late myopia (CS1, PROS1, C1QB and CFD) and late hyperopia (SERPING1, C1QA, CRP, C7 and CFD). In addition, MASP1 (from the lectin pathway) was differentially-expressed in a growth-specific manner (down-regulated in late myopia and up-regulated in late hyperopia). These findings are in line with those of previous targeted studies that identified up-regulation of serum complement markers in patients with pathological myopia^38^ and increased C1q and C3 protein expression in the sclera of myopic guinea pigs^39^. Exploratory transcriptome studies have also implicated C7^13^, CFI^15^, and C3^10^ in animal myopia models. There is comparatively little evidence implicating the complement system in hyperopia, with only one transcriptome study identifying up-regulation of C7 in the chick LIH model^13^. Thus, to our knowledge, the present meta-analysis findings provide the first evidence for the involvement of multiple complement pathway genes in hyperopia.

It has previously been suggested that the complement system may promote ECM remodeling in the sclera during myopia induction^39^. The present findings are consistent with this notion, though the up-regulation of complement genes in both growth conditions at the later time-point suggests that any complement-mediated ECM remodeling is unlikely to play a causal role in directional ocular growth (at least in retina/RPE/choroid). On the other hand, our analysis did link complement with a small network of genes that were differentially-expressed in a growth-specific manner. This network was composed primarily of genes involved in immune-mediated ECM remodeling, and included connections with many intermediate genes that have been strongly linked to myopia in previous targeted studies (e.g. MMP2, TGFB1, TGFB2, FGF2, INS, and IGF2; see Supplementary Table S6). These previous findings lend support to the relevance of the immune-ECM network as a whole, suggesting the need for further research into the role of the lesser explored members (i.e., PTX3, MASP1, NOV, MBL2, C1QA, AP-1, FCN1, and FCN2) in directional ocular growth and refractive change.

In addition to complement pathway and structural genes, five genes encoding mitochondrial ribosomal proteins and one gene encoding a subunit of mitochondrial complex I (NDUFS5) were up-regulated in late myopia. These findings are in accordance with previous omics studies showing enrichment of genes and proteins from the oxidative phosphorylation pathway in chick and mouse models of myopia^13, 40^. Together with this past research, our findings are suggestive of an increase in mitochondrial oxidative phosphorylation in late myopia similar to that seen in some cancers^41–43^. Such an up-regulation of oxidative phosphorylation in myopic eyes is theoretically plausible given the compromised choroidal vasculature^44^, and the increased need for energy to fuel cell growth^9, 45–47^ and maintain ionic gradients^48–50^. It must be noted, however, that two of the mitochondrial ribosomal genes (MRPS21 and MRPL2) were also up-regulated in late hyperopia. This finding is contrary to our previous reports of bidirectional expression of metabolic pathways in early myopia and hyperopia^13^, presumably reflecting the restoration of normal growth rates as refractive compensation to positive lenses is achieved at later time-points.

If oxidative phosphorylation was up-regulated in late myopia and hyperopia, concurrent differential expression of antioxidant defense genes would be expected to compensate for associated changes to the production of reactive oxygen species through electron leak^51, 52^. Such expression changes were evident, particularly in the late myopia dataset where a wide range of genes linked with oxidative stress were either up-regulated (GPX1, GSTA3, MT-4, APOA1, OTUB, PTGDS, HSPB1, HSPB2) or down-regulated (XHD, SLC40A1, TF, SOD3, HPGDS, BCMO1). In addition to ‘classic’ antioxidant enzymes (SOD3, GPX1, GSTA3)^53, 54^, these genes included those involved in regulation of the p53 stress pathway (OTUB)^55^, lipid peroxidation (APOA1)^56, 57^, labile iron levels (XHD, SLC40A1, TF)^58–60^, intracellular zinc homeostasis (MT4)^61^, and arachidonic acid metabolism (PTGDS, HPGDS)^62^.

The factor/s driving these highly similar expression profiles across myopic and hyperopic groups require further investigation. The strong dissociation of responses at early and late time-points certainly suggests that most of the non-specific expression changes (i.e., those in structural, complement-coagulation, metabolic, and oxidative stress pathways) occurred secondary to other processes. It is also important to highlight that the representation of growth non-specific responses may have been increased by the meta-analysis design, which combined studies using relatively heterogeneous methodologies (e.g. different tissue compositions and exact optical manipulations). This choice was intended to identify genes showing differential expression across a broad range of myopia-inducing or hyperopia-inducing conditions, thus generating a better understanding of the generalized transcriptome responses underlying each type of refractive error. The improved concordance of our meta-analysis results with findings from human GWAS and animal proteomics studies of refractive error suggests that this approach was, at least in part, successful. However, this design presumably also biased the results towards identification of genes that respond to varied stimuli (e.g. stress-related genes) and genes that respond to the non-specific effects of lens-wear (e.g. blur or heat under the goggle)^63^.

As mentioned above, human orthologs of sixteen of the genes differentially-expressed in our meta-analysis fall in close proximity to GWAS refractive error peaks in humans. To date, debate surrounding the relevance of animal models to human myopia has primarily focused on similarities and differences in visual experience, refractive development and anatomy (for review see refs 4, 64–68). Our findings contribute to an additional small but growing body of evidence for molecular similarities between humans and animal models^21, 65, 69–73^, made possible by the recent availability of large-scale refractive error GWAS^74^. The two induction time-points in our meta-analysis were roughly proportionally represented in the overlap with human GWAS candidate genes. This is notable because, although some of the overlapping data originated from case-control GWAS of pathological myopia, many of the commonalities were with the CREAM^75^ and 23&Me^76^ data which identified loci associated with spherical equivalent and myopia age of onset, respectively, in large cohorts. Thus, although the chick studies included in our meta-analysis involved the rapid induction of a moderate (early time-point) to high (late time-point) degree of refractive error, it seems that findings in this model may be useful for understanding the broad spectrum of refractive errors present in human populations.

Many of the genes and pathways implicated in our meta-analysis have also been previously associated with AMD, CNV, and/or cataract in humans. Extracellular matrix remodeling^77–79^, activation of immune/inflammatory processes (particularly within the complement system)^80–86^, and oxidative stress^87–91^ have all been implicated in AMD and CNV pathogenesis. Oxidative damage is also thought to be a major factor in the pathogenesis of age-related cataract^92, 93^. More commonalities with human pathology genes were evident at the late (relative to the early) induction time-point. This is consistent with the greater risk of pathological complications in humans with high degrees of refractive error^27–30^, as well as ultrastructural studies suggesting that extreme occlusion-induced myopia can lead to pathological complications in chick^49, 94–96^. In this context, it is plausible that the observed commonalities could provide some insight into the molecular mechanisms underlying the progression from refractive errors to secondary pathologies. For example, up-regulation of oxidative defense genes in the present study is consistent with the theory that myopia may precipitate cataract development by increasing the generation of toxic lipid peroxidation by-products in the retina^27, 97^. However, it must be noted that the overlap of myopia and hyperopia genes with secondary pathologies was not exclusive to disorders for which they are a risk factor. For example, there is no reported association between myopia and AMD^27^, yet both early and late myopia conditions overlapped significantly with the genes associated with sub-clinical pre-AMD and AMD itself. Similarly, to our knowledge, hyperopia has not been linked with CNV in human populations despite the two common gene associations demonstrated here.

This lack of specificity could reflect several factors. Due to current data availability, the disease-associated gene lists could not be sourced from studies specifically investigating the pathological complications of refractive errors. The CNV genes, for example, were associated with ‘wet-AMD’ type CNV rather than myopic CNV. Similarly, the use of data from human genetic linkage and association studies made it difficult to determine whether the fold changes seen in myopic and hyperopic chicks are concordant or discordant with those seen in pathology (the latter possibly conferring a protective effect). Finally, many of the commonalities converged on pathways that displayed growth non-specific expression patterns in the meta-analysis, raising the possibility that they reflect responses to side-effects of lensing in the chick model (e.g. increased heat) rather than responses specifically related to changes in ocular growth and refractive state. Irrespective of the many questions raised, such extensive overlap between pathology-associated genes and the genes differentially-expressed in an animal model of relatively mild environmental perturbation is remarkable, and highlights optical-induced refractive error in chick as a potentially useful model for studying ‘pathology-type’ molecular responses of relevance to a range of human ocular disorders.

In summary, our rank product meta-analysis implicated highly similar genes in chick myopia and hyperopia models. The results provide novel evidence for transcriptional activation of the complement system during both myopia and hyperopia induction, and confirm existing literature implicating cell signaling, mitochondrial metabolism, and structural processes in refractive error. Further comparisons to previous research demonstrate improved concordance with human GWAS and animal proteomics studies of refractive error, and extensive similarities with the genes implicated in sight-threatening ocular pathologies.

## Methods

### Transcriptome study inclusion criteria

Datasets were included in the meta-analysis if they used a commercially available microarray platform or RNA-sequencing to compare gene expression in chick retina, RPE and/or choroid during optically-induced myopia or hyperopia induction, and normal development. Five studies meeting these inclusion criteria on 02/01/2017 (Table 1) were downloaded from Gene Expression Omnibus (GEO)^98^.

**Table 1:** Transcriptome studies meeting inclusion criteria for meta-analysis

Author	Year	GEO	Platform	Tissue	Experimental Cond	Control Cond	Samples Per Cond
McGlinn	2007	GSE6543	Affymetrix	Ret/RPE	Occlusion Myopia at 6 h & 3d	Fellow NL	6
Schippert	2008	GSE11439	Affymetrix	Ret	LIH (+6.9 D) at 1d	Separate NL	4
Ashby	2010	GSE17758	Affymetrix	ACL	LIM (−7 D) & LIH (+7 D) at 1d	Separate NL	3
Stone	2011	GSE24641	Affymetrix	Ret/RPE	LIM (−15 D) and LIH (+15 D) at 6 h & 3d	Fellow NL	6
Riddell	2016	GSE78042	Illumina HiSeq	Ret/RPE/Ch	LIM (−10 D) and LIH (+10 D) at 1d, 2d & 3d	Separate NL	3–4

Abbreviations are as follows: Ret = Retina, RPE = Retinal Pigment Epithelium, ACL = Amacrine Cell Layer, Ch = Choroid, Cond = Condition, LIM = Lens-Induced Myopia, LIH = Lens-Induced Hyperopia, d = days, h = hours, NL = No Lens.

### Data preprocessing

Microarray CEL files were RMA background corrected and quantile normalized using the affy package (v1.52.0)^99^. Probe annotations were updated (Affymetrix release 36, 04/13/16) and, because down-stream processes required a single measure for each gene, probe identifiers were then collapsed to the gene level by retaining only the probe with the highest average expression. This method was chosen as it provides good between-study consistency^100^. RNA-seq counts per million (CMP) data from Riddell *et al*.^13^ were imported into R and log_2_ transformed with an offset of 1 (i.e., log_2_(CMP +1)). The pre-processed microarray and RNA-seq datasets were merged into a single matrix containing only the 10,062 genes measured in all experiments, and then further divided into four conditions representing early (≤1 day) and late (>1 day) myopia and hyperopia induction (as shown in Table 2).

**Table 2:** Data included in each meta-analysis.

Meta-analysis	Author	Date	Details	Samples
Early myopia	McGlinn	2007	6 h occlusion myopia and matched controls	12
	Ashby	2010	1d LIM and matched controls	6
	Stone	2011	6 h LIM and matched controls	12
	Riddell	2016	1d LIM and matched controls	7
	**Total samples in the early myopia meta-analysis: 19 experimental**, **18 controls**			
Late myopia	McGlinn	2007	3d occlusion myopia and matched controls	12
	Stone	2011	3d LIM and matched controls	12
	Riddell	2016	2d LIM and matched controls	7
	Riddell	2016	3d LIM and matched controls	8
	**Total samples in the late myopia meta-analysis: 20 experimental**, **19 controls**			
Early hyperopia	Schippert	2008	1d LIH and matched controls	8
	Ashby	2010	1d LIH and matched controls	6
	Stone	2011	6 h LIH and matched controls	12
	Riddell	2016	1d LIH and matched controls	7
	**Total samples in the early hyperopia meta-analysis: 17 experimental**, **16 controls**			
Late hyperopia	Stone	2011	3d LIH and matched controls	12
	Riddell	2016	2d LIH and matched controls	7
	Riddell	2016	3d LIH and matched controls	8
	**Total samples in the late hyperopia meta-analysis: 14 experimental**, **13 controls**			

Abbreviations are as follows: LIM = Lens-Induced Myopia, LIH = Lens-Induced Hyperopia, d = days, h = hours.

### Rank product meta-analysis

Differentially-expressed genes (DEG) were identified using the Bioconductor *RankProd* package (v.3.0.0)^25, 26, 101^. Genes with a pfp (percentage of false positive predictions) of <0.05 were considered differentially-expressed. This pfp cut-off is theoretically equivalent to a false discovery rate of 5%^101^. When a single study contained multiple experimental conditions (i.e., multiple time-points within early or late categories; see Table 2), the expression values from each condition were considered as a separate dataset for the purpose of the meta-analysis.

### Evaluation of the meta-analysis results

#### Correspondence at the top plots

Correspondence at the top (CAT) plots^102^ were used to evaluate the contribution of each dataset to the meta-analysis results. A rank product analysis of differential gene expression in each individual dataset was conducted as described above. The results of these individual study analyses and the meta-analysis were ordered by the rank product statistic (RP/Rsum), and the proportion of individual study results present in the meta-analysis results was plotted as a function of list size.

#### Comparison with previously published omics data

The meta-analysis results were compared with previously published omics data in two stages. Firstly, they were compared with the results of the individual studies that were meta-analyzed to identify the number of replicated and unique gene findings. Then, both the individual study results and the meta-analysis results were compared to the results of human GWAS and animal proteomics studies to assess whether the meta-analysis improved concordance with these broader omics data types. To facilitate these latter cross-species/cross-platform comparisons, DEG from the meta-analysis and from Riddell *et al*.^13^ were converted to human ortholog Ensembl Gene IDs as described previously^21^. All other data for the comparisons were derived from our previous publication^21^ (i.e., human orthologs of differentially-expressed proteins from proteomics studies, DEG and human orthologs of DEG from the original transcriptome studies, and genes within 500 Kilobases (Kb) and 250Kb of GWAS refractive error and axial length loci). The statistical significance of common findings across each list of genes was then assessed using the R software package *GeneOverlap* (version 1.10.0) as described previously^21^.

### Pathway over-representation analysis

To facilitate interpretation, DEG from each condition were tested for over-representation of Reactome pathways using ConsensusPathDB (v.31)^103^. The manually-curated Reactome database was chosen for use because it is currently one of the most complete open source pathway repositories^104^. The 10,062 genes analyzed were used for the background list, and gene sets with an FDR q-value of <0.05 with ≥3 members were considered over-represented. Over-represented pathways with similar biological functions and identical gene contributions (i.e., redundant results) were collapsed into a single annotation, and the non-redundant pathway findings were then visualized as bubble plots using the R GOplot package^105^.

### Testing overlap between meta-analysis conditions

Gene and pathway findings across the four conditions (early and late myopia and hyperopia induction) were compared using the R software package *GeneOverlap*. The statistical significance of overlapping findings was calculated relative to the 10,062 genes included in the meta-analysis (for DEG) or the 1786 Reactome pathways included in the over-representation analysis (for pathways). Pairwise overlaps between conditions were visualized as heat maps showing the intersection, *Benjamini-Hochberg* adjusted *p-*value, and Jaccard Index. The overlap analysis identified 15 genes that were differentially-expressed in a growth-specific manner (i.e., concurrently up-regulated in myopia and down-regulated in hyperopia, or visa versa). These genes were further investigated using ConsensusPathDB’s Induced Network Module Analysis that connects seed genes through different types of interactions^106^. The analysis was conducted using high and medium confidence filters, genetic, biochemical & gene regulatory interactions, and an intermediate node Z-score threshold of 15.

### Testing overlap with secondary ocular pathologies

Author curated lists of genes associated with AMD, CNV, POAG, and cataract in humans (either via genetic linkage/association or RNA/protein expression changes) were sourced from recent publications as follows. Genes associated with AMD and/or drusen composition were sourced from Table S2 of Newman *et al*.^79^ Genes linked to CNV were sourced from Supplementary Material I of Zhang *et al*.^86^ Genes associated with POAG and its endophenotypes were sourced from Supplementary Table S1 of Iglesias *et al*.^107^ Genes associated with cataract were sourced from Cat-Map^108^ on 2017/02/26. In addition, a large list of genes potentially associated with sub-clinical pre-AMD (i.e., AREDS level 2) were sourced from Table S3 (disease module MD2) of Newman *et al*.^79^ Disease-associated gene lists were converted to Ensembl Gene IDs using BioMart (Ensembl release 86; Homo sapiens GRCh38.p7), and compared to human orthologs of the genes differentially-expressed in the early and late myopia and hyperopia meta-analyses. These comparisons were made using the GeneOverlap package as described previously^21^. The results were visualized as chord diagrams using the R *GOplot* package^105^, and overlapping genes were tested for over-representation of Reactome pathways as described above.

### Data availability

The datasets analysed during the current study are available in the GEO repository, www.ncbi.nlm.nih.gov/geo/. GEO accession numbers are provided in Table 1.

## Electronic supplementary material

Supplementary Tables S1-S7
